# Routine MRI findings of the asymptomatic foot in diabetic patients with unilateral Charcot foot

**DOI:** 10.1186/1758-5996-2-25

**Published:** 2010-04-22

**Authors:** Ludger W Poll, Ernst A Chantelau

**Affiliations:** 1 Department of Radiology, Berufsgenossenschaftliche Unfallklinik Duisburg GmbH, Großenbaumer Allee 250, 47249 Duisburg, Germany; 2 Holthorster Weg 16, 28171 Bremen, Germany

## Abstract

**Background:**

Imaging studies of bones in patients with sensory deficits are scarce.

**Aim:**

To investigate bone MR images of the lower limb in diabetic patients with severe sensory polyneuropathy, and in control subjects without sensory deficits.

**Methods:**

Routine T1 weighted and T2-fat-suppressed-STIR-sequences without contrast media were performed of the asymptomatic foot in 10 diabetic patients with polyneuropathy and unilateral inactive Charcot foot, and in 10 matched and 10 younger, non-obese unmatched control subjects. Simultaneously, a Gadolinium containing phantom was also assessed for reference. T1 weighted signal intensity (SI) was recorded at representative regions of interest at the peritendineal soft tissue, the tibia, the calcaneus, and at the phantom. Any abnormal skeletal morphology was also recorded.

**Results:**

Mean SI at the soft tissue, the calcaneus, and the tibia, respectively, was 105%, 105% and 84% of that at the phantom in the matched and unmatched control subjects, compared to 102% (soft tissue), 112% (calcaneus) and 64% (tibia) in the patients; differences of tibia vs. calcaneus or soft tissue were highly significant (p < 0.005). SI at the tibia was lower in the patients than in control subjects (p < 0.05). Occult traumatic skeletal lesions were found in 8 of the 10 asymptomatic diabetic feet (none in the control feet).

**Conclusion:**

MR imaging did not reveal grossly abnormal bone marrow signalling in the limbs with severe sensory polyneuropathy, but occult sequelae of previous traumatic injuries.

## Introduction

Neuro-osteoarthropathy of the feet, i.e. the Charcot foot in diabetes, is believed to be caused either by repeat traumatisation of an injured insensitive foot [1,2], or by a hypothetical neurogenic bone dystrophy [2,3], or both. Hence, the „diabetic“ Charcot foot might be caused by a specific osteopathy, or by an „abnormal“ reaction of the „diabetic bone“ to simple mechanical stress [4]. While it is undisputed that a most severe loss of protective sensation, and of pain sensation in particular [5], is a necessary prerequisite for a Charcot foot to develop, bone abnormalities consistent with a neuropathic osteopathy so far have not been demonstrated convincingly. Direct histomorphometric bone analyses-apart from animal studies- are lacking; there are only few, albeit crude, histopathological data from human studies available [6]. Studies assessing bone mineral density (BMD) in diabetic neuropathy have yielded conflicting results, due to problems with patient selection and methodology [2]. Foot BMD is increased in Type-2 diabetes mellitus (most likely in relation to the increased prevalence of overweight in this patient population), whereas in Type-1 diabetes mellitus, it is slightly reduced (-5% compared to control subjects), most likely due to insufficient IGF-1 generation in this population [7]. Bone morphology studies with X-ray and other imaging techniques have rarely been published [8-11]. We therefore set up an MR imaging study of the calcaneal and tibial bone in patients with most severe diabetic neuropathy, i.e. in diabetic patients with a history of unilateral, healed Charcot foot, and in matched and non-matched healthy control subjects. In extension of our previous work on symptomatic Charcot feet [12], we used the contralateral (asymptomatic) non-Charcot foot for the present investigation.

## Subjects and methods

### Patients

Diabetic patients (n = 10) with unilateral, healed Charcot-foot, chronic inactive for at least 1 year, were recruited from the outpatient diabetic foot clinic at the Heinrich-Heine-University of Düsseldorf. All patients displayed severe peripheral sensory polyneuropathy (vibration sensation at the first metatarsal head < 4/8 grades by Rydel-Seiffer tuning fork [13]). At the time of the study they were free of active foot ulceration, relapse or re-activation of the Charcot-foot. Two of the patients had endstage renal disease with kidney transplantation (n = 1), or hemodialysis (n = 1).

### Control subjects

Healthy matched controls (n = 10) were recruited from relatives of the staff of the traumatology clinic in Duisburg-Buchholz (Berufsgenossenschaftliche Unfallklinik Duisburg GmbH/Germany). They were eligible if they did not have diabetes mellitus (according to self-reported absence of antidiabetic medication), and if they matched to the patients' age (+/- 5 years), body mass index (+/- 1 kg/m^2^), and gender.

Another group (n = 10) of younger (60 years and less) and less overweight (BMI <28 kg/m^2^) healthy unmatched control subjects was also studied.

The clinical characteristics of the participants are summarised in Table 1.

**Table 1:** clinical characteristics and MRI findings

	diabetic patients	matched controls	non-matched, non-diabetic, younger controls
number(f/m)	10(5/5)	10(5/5)	10(4/6)
diabetes type-1, n	3	0	0
diabetes type-2, n	7	0	0
duration of diabetes, yrs	24(20-27)	0	0
age, yrs	59(53-65)	58(52-64)	45(37-53)
body mass index, kg/m^2^	29(26-33)	28(26-30)	25(23-27)
polyneuropathy, n	10	0	0
			
MRI skeletal damage, n	8	0	0
			
T1 weighted SI in% of phantom SI			
			
..soft tissue	102(80-126)	104(99-109)	105(101-110)
..calcaneus	112(100-123)	102(96-108)	107(102-112)
..tibia*	64(49-80)**	85(76-93)	82(77-86)

SI = signal intensity; means (95% confidence intervals),*p < 0.005 versus calcaneus and soft tissue, respectively;** p < 0.05 versus tibia in controls

Body mass index (BMI) was calculated using self-reported weight (kg) and height (m); a BMI > 28 kg/m^2 ^was assumed to indicate relevant overweight.

### Examinations

Routine MR examinations were performed on a 1.5 Tesla superconducting magnet (Magnetom Avanto, Siemens). Subjects were examined in supine position, placing the feet first into the gantry. All examinations were performed with a head-neck surface coil. Contrast media was not applied to the study participants. Each foot was scanned in sagittal view using T1-weighted turbo-spin-echo (TSE) sequences (TR: 580, TE: 15) with a slice thickness of 3 mm. Paracoronal T1-weighted TSE-sequences were acquired parallel to the midfoot through tibia, calcaneus und talus. Sagittal T2-fat-suppressed-STIR-sequences (TR: 3200, TE: 27, TI 160 msec) were also acquired [14]. Paracoronal T1-weighted TSE-sequences und STIR-sequences were acquired parallel to the midfoot and an axial T2-weighted-fat-suppressed sequence was performed through tibia, calcaneus und talus. Total examination time was about 40 minutes. All MR examinations had diagnostic quality and were well tolerated by all patients; they were carried out by an experienced musculoskeletal radiologist (L.W.P.).

To account for day-to-day variations in the sensitivity of the coil, a phantom prepared with contrast media (Gadolinium) was repeatedly subjected to MRI together with the feet; its signal intensity - assessed as described below- was highly reproducible (coefficient of variation 3.7%).

### Signal intensity

**Figure 1:**
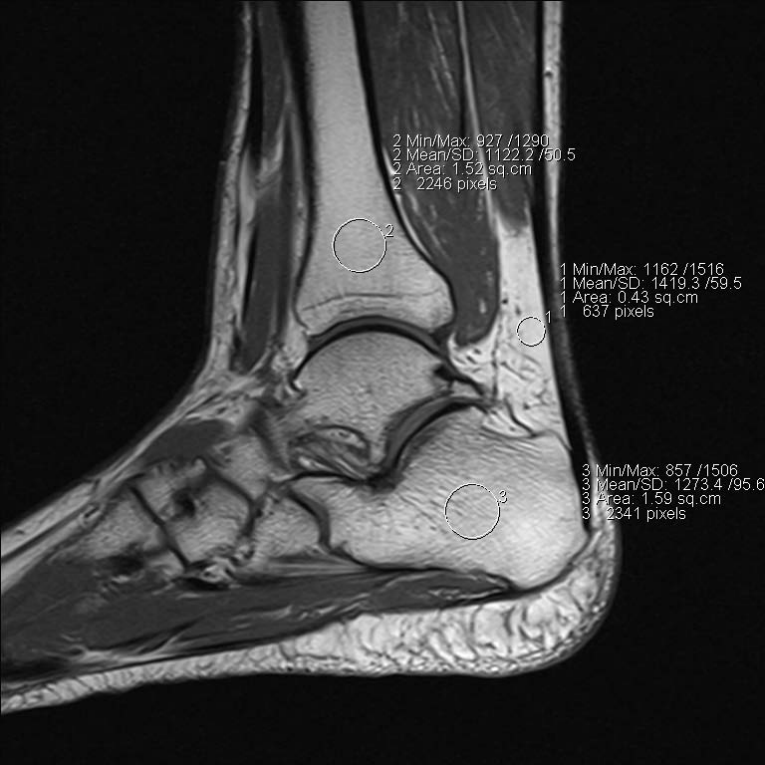
**Sagittal T1-TSE-Sequence of a right foot, with regions of interest (ROI) deliberately placed in peritendineal soft tissue (1), distal tibia (2), and calcaneus (3)**.

Signal intensity was assessed on sagittal T1-weighted TSE sequences. To this end, on the main console of the magnet region-of-interest (ROI) measurements were drawn electronically. Circular regions of interest of 0.5-1.5 cm diameter - as appropriate- were positioned at the distal part of the tibia, at the centre of the calcaneus, at the adjacent soft tissue underneath the achilles tendon (Figure 1), and at the contrast media container. Signal intensity (expressed in arbitrary units) assessments were highly reproducible (coefficient of variation < 4%), irrespective of the ROI size. Assessments were performed in duplicate one week apart with repositioning of the ROIs, with a third assessment being made if the two means differed by 5% or more, and the means were averaged. High SI indicates fat, low SI indicates water [15].

### Calculations

Bone and soft tissue signal intensity was analysed as percentage of the mean signal intensity of the Gadolinium phantom.

### Ethics

All subjects gave written informed consent. The study was approved by the ethical committee of the medical faculty of the Heinrich-Heine-University of Düsseldorf/Germany (Study Nr 2717).

### Statistics

Data are presented as means with 95% confidence intervals, and processed for descriptive purposes only. Student's t-test was applied with p < 0.05 as level of significance.

## Results

In the matched control subjects, SI at the peritendineal soft tissue, the calcaneus, and the tibia, respectively, was 104%, 102% and 85% of that at the phantom, compared to 105%, 107% and 82% in the non-matched controls, and 102% (soft tissue), 112% (calcaneus) and 64% (tibia) in the neuropathic patients. SI was significantly lower at the tibia (than at the calcaneus or peritendineal soft tissue) in all three subject groups (p < 0.005); SI at the tibia was lower in the neuropathic patients than in control subjects (p < 0.05) (see Table 1). In 8 of the 10 clinically asymptomatic feet of the neuropathic patients, occult residual traumatic lesions were found (stress injuries in 2 cases, healed ankle fracture in 1 case, healed metatarsal shaft fracture in 1 case, ruptured Achilles tendon in 1 case, osteochondrosis dissecans in 2 cases, activated arthrosis in 2 cases); in 6 of these cases, injuries (e.g. painful events) were not recalled by the patients. Non-traumatic lesions were found in 6 cases (bone cyst (n = 4), and calcaneal spur (n = 2)). None of the control subjects displayed any skeletal abnormalities.

## Discussion

The present data show that routine MR imaging [16] may not reveal grossly abnormal bone marrow signalling in the limbs with severe sensory polyneuropathy, but occult sequelae of previous traumatic injuries.

T1 weighted signal intensity at the bone-indicative of marrow fat [15,17] - was generally lower at the distal tibia than at the calcaneus (p < 0.005). Hence, the relative content of fat seems to be greater in the calcaneus than in the tibia. Interestingly, these findings parallel differences in bone mineral density (BMD) obtained by dual X-ray absorptiometry: BMD is only half as much in the calcaneus than in the femur (approximately 0.5 g/cm^2 ^versus 0.9 g/cm^2^)[18]. BMD at the foot skeleton may be similar in subjects with and without diabetic polyneuropathy [9,18,19]. BMD at the tibia (approximately 1.0 g/cm^2^) may be similar in subjects with and without diabetic polyneuropathy and a history of unilateral Charcot foot [20]. The signal intensity at the tibia was significantly lower in the patients than in the controls; we have no explanation for this.

The present study found occult traumatic bone injuries in 80% of the asymptomatic feet in the diabetic patients with polyneuropathy, although these had not been reported by the patients. In general, recent traumatic events (from ankle sprains, hitting objects in the living room, or overuse [21]) are rarely recalled precisely by elderly subjects [22], and less so by subjects with sensory deficits. A MRI-based study in asymptomatic feet of patients with peripheral sensory nerve dysfunction from leprosy has found residual lesions of prior traumatic injuries in 4 out of 6 of cases (67%) [11], whereas a previous X-ray based study has observed such lesions in 22% of 54 asymptomatic feet with diabetic polyneuropathy [8]. MRI is more sensitive than X-ray to detect traumatic skeletal lesions [23]. Therefore, the higher prevalence of traumatic lesions in MRI-based studies may represent the true prevalence of traumatic injuries (e.g. from sprains, or stress impacts) in asymptomatic feet of patients with peripheral sensory nerve dysfunction. Recent epidemiologic studies seem to support this assumption: diabetes mellitus of long duration is an established risk factor for foot fractures [24]. Moreover, increased foot loading e.g. from obesity increases the risk of foot fractures [25], and of Charcot-foot deformities in diabetic neuropathy in particular [26,27].

Our study has strengths and weaknesses. Its strength is the fact that-to the best of our knowledge-it is the first study to assess bone morphology in asymptomatic feet with diabetic polyneuropathy by MRI. It is a weakness, however, that the sample size is only small, that self-reported data were used, and that the patient population was rather heterogeneous (2 of 10 patients had chronic kidney failure, 3 of the 10 patients had type-1 diabetes mellitus). Hence, our study sample was not appropriate to draw definite conclusions. Moreover, only routine MRI-techniques were employed that do not correlate well with established surrogate parameters of bone strength [28,29]; more specific MRI techniques might provide additional insights into the bone structure in diabetic neuropathy [30-33].

In conclusion, routine MR imaging did not reveal grossly abnormal bone marrow signalling in the limbs with severe sensory polyneuropathy, but a high prevalence of occult sequelae of previous traumatic injuries. According to these preliminary observations, a hypothetical endogenous osteopathy is unlikely. More specific MRI analyses, e.g. by MR histomorphometry are warranted to corroborate these findings in patients with diabetic polyneuropathy.

## Conflict of interests

The authors declare that they have no competing interests.

## Authors' contributions

LWP did the study design, the measurements, data analysis, and drafting and writing of the manuscript. EAC recruited the patients and participated in the analysis of the data and the writing of the paper. Both authors read and approved the final manuscript.
